# Advancing maternal survival in the global context: are our strategies working?

**DOI:** 10.1186/1471-2458-13-689

**Published:** 2013-07-29

**Authors:** Omar A Khan, Richard Derman, Nancy L Sloan

**Affiliations:** 1 Community Health & Preventive Medicine and Director, Global Health Residency Track, Department of Family & Community Medicine Christiana Care Health System, 3506 Kennett Pike/PMRI, Wilmington, DE 19807, USA; 2 Department of Family & Community Medicine, Jefferson Medical College, Philadelphia, PA 19107, USA; 3 Marie E. Pinizzotto Endowed Chair of Obstetrics and Gynecology and Director, Center for Women’s and Children’s Health Research, Christiana Care Health System; Professor, Obstetrics and Gynecology, Thomas Jefferson University, 4755 Ogletown-Stanton Road Suite 1903, Newark, DE 19713, USA; 4 Epidemiologist, Department of Obstetrics & Gynecology, Christiana Care Health System, Department of Population & Family Health; Columbia University School of Public Health, New York, NY, USA

**Keywords:** Maternal health, Postpartum hemorrhage, Child mortality

## Abstract

There have been significant gains in improving maternal mortality over the last two decades. Researchers have suggested a variety of interventions and mechanisms to explain these improvements. While it is likely that much of what has been done in research and programs has contributed to this decline, the evidence regarding what works in the settings in which women deliver continues to face many challenges. We review the evidence for these improvements and suggest that there remain areas to focus on, particularly the births which currently take place in an unsupervised or substandard environments. We highlight the main areas where more evidence is needed, and end with a call to determine which of our interventions seem to have the most benefit; which do not; and where to invest future resources.

## Background

There have been great advances in maternal survival between 1990 and 2010 [1]. In 1990, the WHO estimated 543,000 women each year experienced pregnancy-related deaths, and declined to 287,000 by 2010, 99% of which occur in developing countries. This trend is supported by other estimates [2]. Theories have been proposed to explain these achievements [3]; but the theories may or may not be based upon sound research, and unaddressed issues remain [4-6].

## Main text

What we do know is that many maternal deaths occur because they are not receiving timely, quality intervention [1,2,7]. Unlike hospital settings that provide quality services, many women, including some of those delivering at home or at institutions providing substandard care, do not receive the benefits of appropriate management. This may include provision of additional uterotonics, manual removal of the placenta, hysterectomy and/or transfusion for continued postpartum bleeding, Caesarean section for dysfunctional labor, magnesium sulfate for eclampsia, antibiotic treatment for infection and nutritional supplementation.

The questions about what caused the improvements in maternal survival are justifiable. Many have argued they are due simply to improving skilled birth attendance or institutional deliveries, even in settings where such attendance often cannot or does not provide the essential obstetric management required [8,9]. However, provision of skilled attendance alone, outside the institutional setting, has proven less effective than hoped [10], while quality improvement mechanisms have accomplished great achievements in institutional service provision [11-13]. Management without sufficient or adequately trained personnel, medication and infrastructural resources, much less a hygienic environment, may not effectively manage some or many of the conditions that cause maternal mortality [14].

Improving many factors may be necessary, such as permitting community-based attendants to provide oxytocin injections to prevent postpartum hemorrhage, and agents such as misoprostol that effectively prevent postpartum hemorrhage [15]. Improving the continuum of care and timely use of institutional care may require roads, transportation, better education and the desirability of care (including its quality) all of which may require economic development and societal beneficence [16]. Improving the quality of care requires more than supplies, equipment and training, but also requires establishing processes that integrate understanding patients needs, engagement of health providers, monitoring and reviewing data regarding how care processes function within the delivery system, and ongoing modification of both the processes and the systems to improve care and outcomes [12,17].

## Discussion

### The knowledge gap: (not) knowing what works

Millions of dollars have been spent over the past decades to improve maternal survival, yet we still do not know if the observed improvements are attributable to the interventions we are conducting, or due to other factors, such as additional supportive services and/or overall economic improvement^12^. Ten years ago, we suggested that it would be necessary and wise to directly assess the impact of our interventions on maternal mortality rather than solely evaluating surrogate measures [18]. For years, the conventional wisdom held that measuring maternal mortality was just too difficult and expensive [19]. In our efforts to improve infant survival, it took decades to recognize that prematurity and infection were the major etiology of most infant mortality in low birth weight babies and that efforts solely to improve caloric intake were ineffective in improving survival [20]. Similarly, it has taken decades to suggest that measuring maternal mortality may be necessary and is worthwhile [13,21].

Only a handful of methodologically sound studies to demonstrate the effectiveness of priority interventions delivered in substandard environments have been conducted [22-24]. The NIPPS study that found vitamin A supplementation nearly halved maternal mortality rates in Nepal (in a setting with a high prevalence of moderate-severe vitamin A deficiency) was met with great controversy. Arguments were made that the reported causes of death were not coherent, even though the causes of death were reported as symptoms by lay people, relatives of the deceased in an area where illiteracy is common and medical diagnostic capacity is virtually absent [25]. A large replication trial was conducted in rural Ghana, in an area where the consumption of red palm oil is common, with the stated objective of disproving that vitamin A supplementation could reduce maternal mortality [26]. As with a replication study conducted in Bangladesh [27], however, the low prevalence of moderate-severe vitamin A deficiency undercut the studies’ potential to find an association between supplementation and survival. An extremely large study, but with inadequate and incomplete measurement of the outcome variable, child mortality, proposes to overturn policies and programs that have saved millions of children’s lives [28,29]. Some have proposed that the millions spent on programs to prevent postpartum hemorrhage have significantly reduced maternal mortality, yet this has not been directly assessed by rigorous research in the settings where there is substandard access to and/or quality of care, where most maternal mortality occurs [30]. The fact that uterotonics are more effective at preventing high levels of blood loss supports the notion that their provision may be preventing maternal mortality^24^. However, we still do not know if the women who actually die of postpartum hemorrhage are those with extremely high levels of blood loss caused by conditions other than uterine atony for which uterotonics may be relatively ineffective and for which other intervention, such as transfusion and/or, hysterectomy are required. In addition, in these developing country, we still do not know if individual interventions alone such as vitamin A supplementation would improve maternal survival in Niger or Afghanistan, where the prevalence of moderate-severe vitamin A deficiency and maternal mortality rates remain high, nor the answers to other important questions such as whether a loading dose of magnesium sulfate provided in a community-based setting will improve mortality from eclampsia.

### The next steps

We know that the expense and complexity of measuring maternal mortality has hindered our efforts to conduct the rigorous research necessary to determine if our interventions are saving women’s lives in settings where care is substandard; and if so, to what extent and how. Household surveillance systems are excellent mechanisms to support research, yet are not the sole means necessary to achieve nearly universal vital events reporting [31]. While household surveillance systems have benefits beyond accurate reporting, including bringing jobs, improving the local economy, and even nutritional status[32-34], household surveys can provide accurate, complete reporting, and with adequate training, supervision and supplies, validated mobile device reporting may become an option [35,36]. Such techniques can allow us to affordably and accurately measure impact on maternal mortality. Whether as researchers, program managers, or donors, we need to rise to the challenge of strengthening (or in some cases, creating) the evidence base to assess our programs.

## Conclusion

We have started asking the question, ‘what do we know’? The answer seems to be ‘less than we think we do’. We suggest that along with doing what we think makes a difference, we need to confirm it and if necessary, change it. We suggest that, above and beyond monitoring and evaluation, rigorous research is necessary to measure the impact of the interventions we promote for scale up on the theory that they may prevent maternal mortality in settings where women typically receive substandard care to understand if they are indeed effective in such settings without coexistent improvement of supportive care. It is critical to determine whether and which of our interventions improves maternal survival in settings with substandard care, and thus reduce maternal mortality where most of it continues to occur.

## Competing interests

The authors declare that they have no competing interest.

## Authors’ contributions

OK, RD and NS conceptualized the paper. OK and NS completed the background review and wrote the first draft; RD coordinated the submission and contributed to all versions. All authors read and approved the final manuscript. OK is the corresponding author.

## Pre-publication history

The pre-publication history for this paper can be accessed here:

http://www.biomedcentral.com/1471-2458/13/689/prepub
